# The Current Accreditation Standards and Future Needs in Medical Education

**DOI:** 10.31661/gmj.v9i0.1901

**Published:** 2020-09-09

**Authors:** Ebrahim Shirzadeh, Nematullah Shomoossi, Ali Tajabadi

**Affiliations:** ^1^School of Medicine, Sabzevar University of Medical Sciences, Sabzevar, Iran; ^2^School of Paramedicine, Sabzevar University of Medical Sciences, Sabzevar, Iran

**Keywords:** Schools, Medical, Quality Improvement, Program Evaluation, Education, Accreditation, Curriculum

## Dear editor,

Current practice the science education, particularly in medical education, is presumably a mirror of its future directions. Khay-Guan (2019) asserts that today’s medical students will carry our values, skills, and our hopes for the profession into the future. In other words, medical education today represents the future of health and medicine worldwide. Historically, ordinary people used to expect Nostradamus (the famous French astrologer and physician predicting future events in his poetic quatrains) to predict the future. However, without relying on poetic predictions, the needs of tomorrow may be addressed differently if realistic perspectives are adopted [1]. An integrated curriculum stressing the competence of career options might include a broader scope of elements in addition to student-focused learning activities [2]. Han *et al*. (2019) suggest four major themes to be included in the curricula: (1) a humanistic perspective on patient safety; (2) primary orientation towards patient-oriented integration and long-term clerkships; (3) moving away from hospitals toward society; and (4) student-led instruction aided bytechnology [3]. However, Blouin and Tekian (2018) stressed moving away from a focus on student outcomes as measures of accreditation and embracing additional markers such as continuous quality improvement (CQI) orientation [4]. An all-inclusive curriculum is, therefore, expected to include common values together with quality assurance; this led to the development of a uniform accreditation scheme, known as WHO/WFME Guidelines for Accreditation of Basic Medical Education (2005), which listed the main elements as mission and aims, instructional plans, assessment of learners, learners themselves (issues other than assessment, such as selection, number, etc.), faculty members, educational resources, program evaluation, supervision and administration, and continuous appraisal of the plans. According to WFME (2020), accreditation is defined as the certification of the appropriateness of medical education programs, and the empowerment of medical schools in the instruction of medical education curricula. Also, the task of accreditation was assigned to governments, or institutions who receive their authority from governmental ministries. Accordingly, a directory of international organizations was developed by WFME in each country to recognize, accredit, or approve medical schools and their programs [5]. Furthermore, the policy of the Educational Commission for Foreign Medical Graduates (ECFMG) (2010) of the USA stated that since 2023, physicians applying for ECFMG Certification should be required to graduate from a medical school which has been accredited by standard routines. The purpose behind the policy was to encourage countries to develop accreditation systems for basic medical education if they had not already taken measures.

 To be concise, current instructional perspectives and accreditation schemes heavily rely on organizational orientations (e.g., mission and objectives), educational elements (e.g., educational program, assessment of students, students recruitment), manpower (e.g., academic staff/faculty), management (e.g., instructional resources, program evaluation, and supervision and administration); only one element on the list of WHO/WFME (2005) signifies ‘continuous renewal’, which is expected to be a hint for keeping up with advances in technology, sociology, and philosophy of learning and teaching. However, human knowledge is speedily expanding edges of science in areas such as information and communication technology, learning management platforms, paper-less education, blended learning, mobile learning, and podcasting, embodied learning, inquiry-based learning, learning through an international language, and cross-cultural communication.

To this list, we may add unpredictable shifts in global demographics in the 21st century due to faculty and student’s mobility [6]. A gap might then appear if the current accreditation standards are considered with the future image of medical education and practice. An example is the pandemic outbreak of COVID-19, which required scientific predictive measures in advance. In short, we may either (1) intend to ‘form the future’ by the current principles of accreditation of medical education, or (2) we have the option of ‘preparing the medical education’ for unpredictable future needs and trends [7]. Even if we successfully cater for the present needs of the field, the future is unpredictable and the influence of developing frontiers of human science (including the psycho-social theories of learning and teaching, global trends analysis and shifts in global demographics) will affect the future of medical education. Future practitioners will succeed if and only if they are trained with the prospective empowering goals. We emphasize ‘prospectively potentiating elements’ in medical education and remind educators to adjust their goals and strategies accordingly, by reforms [8] in the workforce (faculty empowerment and students’ competency orientation) and institutional orientations (including accreditation schemes).

## Conflict of Interest

 Nothing to declare.
